# Zoonotic Transmission of Soil-Transmitted Helminths in a Setting with Close Human–Animal Interaction: A Cross-Sectional Pilot Study from Meghalaya, India

**DOI:** 10.4269/ajtmh.25-0401

**Published:** 2025-11-11

**Authors:** Uniqueky Gratis Mawrie, Malathi Manuel, Peter John Marbaniang, Innangkyntiew Lyngdoh Sangriang, Selvi Laxmanan, Sitara Swarna Rao Ajjampur, Rajiv Sarkar

**Affiliations:** ^1^ Indian Institute of Public Health, Shillong, India;; ^2^ Department of Disease Control, Faculty of Infectious and Tropical Diseases, London School of Hygiene and Tropical Medicine, London, United Kingdom;; ^3^ The Wellcome Trust Research Laboratory, Division of Gastrointestinal Sciences, Christian Medical College, Vellore, India

## Abstract

Soil-transmitted helminths (STHs) remain a major public health concern, especially in low- and middle-income countries. Although *Ascaris lumbricoides*, *Trichuris trichiura*, and hookworms (*Ancylostoma duodenale* and *Necator americanus*) are commonly identified in humans, emerging evidence using molecular diagnostics capable of differentiating morphologically similar ova suggests zoonotic drivers of transmission, particularly in areas with close human–animal interaction. This pilot study investigated human and animal STH infections across nine villages of Meghalaya in the northeastern region of India, using quantitative polymerase chain reaction with species-specific primers. The estimated STH prevalence was 23.3% (95% CI: 17.9–29.9%). Zoonotic potential was evident, with *Ancylostoma ceylanicum* detected in humans (2.8%), and *Ascaris* spp. and *Necator americanus* detected in pigs (64.5% and 3.2%, respectively) and goats (15.2% and 3.0%, respectively). To meet World Health Organization’s target of eliminating STH morbidity by 2030, endemic regions may need to adopt an integrated One Health approach.

## INTRODUCTION

Soil-transmitted helminths (STH), commonly *Ascaris lumbricoides*, *Trichuris trichiura*, and hookworms (*Ancylostoma duodenale* and *Necator americanus*), remain a major public health concern, particularly in low- and middle-income countries. An estimated 1.38 million disability-adjusted life years are attributed to STH globally.^1^ In India, approximately 20% of the population is infected with STH, with the highest burden in the northeastern region (NER).^2^

Molecular studies suggest zoonotic transmission of *Ancylostoma ceylanicum*, *Ancylostoma caninum*, *Ancylostoma braziliense*, and *Trichuris vulpis* from dogs, and *Ascaris suum* from pigs, indicating potential human–animal cross-infection.^3^ However, the occurrence of zoonotic infections in humans may be underestimated due to reliance on microscopy-based methods, which cannot differentiate between morphologically-similar ova of human and animal STH species.

The WHO aims to reduce the prevalence of moderate-to-heavy intensity STH infections to <2% in endemic countries by 2030, primarily through periodic deworming of high-risk groups (preschool and school-aged children, nonpregnant adolescent girls, and women of reproductive age).^4^ However, in the absence of improvements in water, sanitation, and hygiene, the impact of these efforts may be limited, particularly in the presence of untreated reservoirs of infection, including zoonotic reservoirs. Although WHO advocates a One Health approach to address neglected tropical diseases,^4^ its application to STH control remains limited. This pilot study investigates the presence of STH in humans and animals in indigenous villages of Meghalaya, NER of India, where mixed farming practices and close human–animal interactions are common.

## MATERIALS AND METHODS

### Study area and population.

**Figure 1.**
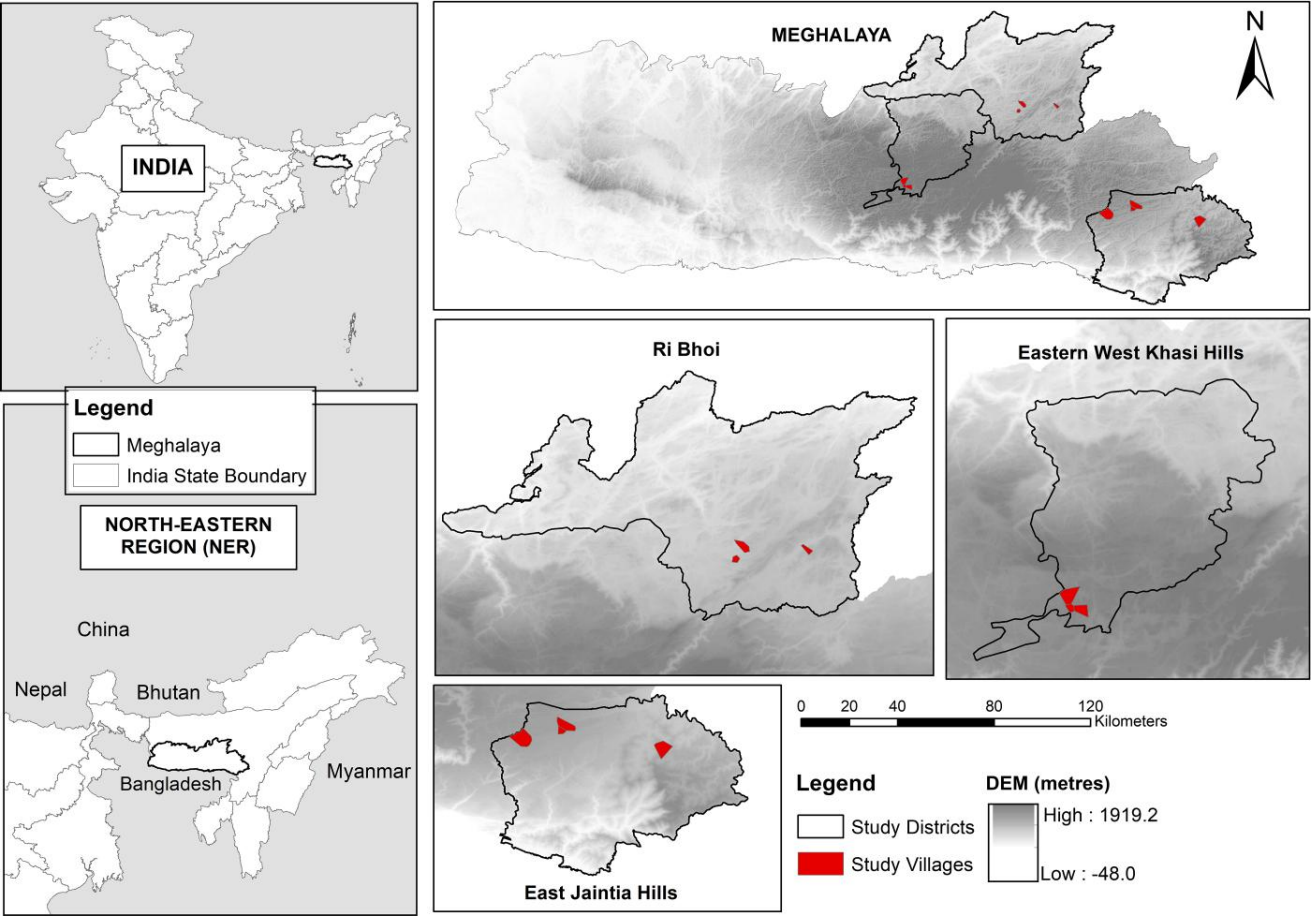
Map of Meghalaya, India, highlighting the study area.

A cross-sectional study was conducted in nine villages across three districts of Meghalaya (Ri Bhoi, Eastern West Khasi Hills, and East Jaintia Hills [Figure 1]), each with unique environmental and climatic characteristics conducive to STH transmission. Meghalaya is inhabited by predominantly rural, indigenous populations with high rates of malnutrition.^5^ An earlier study reported 49% STH prevalence in school-aged children, using microscopy-based methods.^6^ Backyard animal farming is common, with animals reared in close proximity to human dwellings.

Using a multistage sampling approach, one primary health center per district, and three villages were chosen from each primary health center catchment area. Within each village, households were selected systematically. From each household, one child (aged 2–14 years) and one adult (aged ≥15 years) were enrolled. In some households, only one participant was available to provide a sample. In three households, where two members were initially recruited, families requested the inclusion of an additional member. To maintain participation and cooperation, one additional sample was collected in each of these households. Structured interviews were conducted to collect data on demographics, hygiene, animal exposure, and agricultural practices. One fecal sample was collected from each participant, and up to five pooled samples were collected from household-owned livestock (pigs and goats); each pooled sample was treated as a single observation in the analysis. Samples were transported on a cold chain to the Indian Institute of Public Health (IIPH) Shillong within 24 hours, where they were aliquoted into two sterile cryovials (500 mg of stool samples in 95% ethanol) and stored at −80°C until further processing.

### Laboratory methods.

All fecal samples were tested at the Wellcome Trust Research Laboratory, Christian Medical College (CMC), Vellore, India, using established protocols.^7^^,^^8^ For human samples, after bead beating with the Omni Bead Ruptor 96 (OMNI International, Kennesaw, GA), DNA was extracted using the MagMAX Microbiome Ultra Nucleic Acid Isolation Kit on the KingFisher Flex system (Thermo Fisher Scientific, Waltham, MA). For animal fecal samples, DNA was extracted using the SPINeasy DNA Pro Kit with the FastPrep-24 5G homogenizer (MP Biomedicals, Irvine, CA). Extracted DNA was tested by quantitative polymerase chain reaction (qPCR) for *N. americanus, A. duodenale, A. lumbricoides/A. suum, T. trichiura, S. stercoralis, A. caninum*, and *A. ceylanicum* using the QuantStudio system (ThermoFisher Scientific), and previously published primers targeting repetitive sequences.^9^^–^^11^ DNA extraction and qPCR included internal amplification and positive controls, as well as reagent-only controls. A cycle threshold (Ct) ≤40 was considered positive (Supplemental Text 1).

## STATISTICAL ANALYSES

Data were analyzed using R version 4.3.2 (R Foundation for Statistical Computing, Vienna, Austria; https://www.r-project.org/). Continuous variables were summarized using means and standard deviations or median and interquartile range, based on distribution. Categorical variables were described using frequency counts and marginal percentages.

To account for the hierarchical data structure, the survey design parameters were defined using “svydesign” function from the R “survey” package.^12^ Sampling weights were calculated as the inverse probabilities of selection at the village and household levels to generate population-representative estimates. The prevalence estimates and 95% confidence intervals were obtained with the “svydesign” function, with standard errors calculated using the Taylor linearized method. Associations between categorical variables were assessed using χ^2^-tests adjusted for complex survey design.

## RESULTS

### Demographic details.

A total of 297 individuals from 154 households were approached, of whom 278 (93.6%) from 149 households consented, completed the questionnaire, and provided stool samples for testing. Of these, 123 households contributed two participants each (typically one child and one adult), 23 households had only one participant, and 3 households provided stool samples from three participants. Additionally, 126 animal fecal samples (33 from goats and 93 from pigs) were collected from 104 (69.8%) households. Of these, 58 (46.0%) were pooled samples: 38 pooled pig and 20 pooled goat samples. Among the human participants, 61.9% were females and 30.9% were aged 6–14 years. The mean (SD) age of the adult participants (aged ≥15 years) was 37.0 (12.2) years.

Among participants aged 5–18 years, 12.4% were not attending school. Approximately 78% of participants reported having been dewormed in the previous 6 months. Approximately 60.4% of the participating households had at least one member involved in agriculture. Animal ownership was reported in 69.1% of households, primarily pigs (69.6%). Open defecation was reported by 16.1% households, and 72.5% used animal feces as manure.

### STH prevalence in humans.

The overall prevalence of STH infection was 23.3% (95% CI: 17.9–29.9%). *Necator americanus* was the most common species detected (13.8%; 95% CI: 8.7–21.2%), followed by *Ascaris* spp. (7.4%; 95% CI: 4.4–12.2%) and *T. trichiura* (1.5%; 95% CI: 0.4–5.9%). The prevalence of *A. ceylanicum* was 2.8% (95% CI: 1.3–6.0%). *Ancylostoma duodenale*, *A. caninum*, and *S. stercoralis* were not detected.

The STH prevalence by age group was 13.9% (95% CI: 4.8–34.2%) in children aged ≤5 years, 20.8% (95% CI: 8.8–41.9%) in those aged 6–14 years, and 28.2% (95% CI: 20.0–38.3%) in individuals aged ≥15 years. Among females, the prevalence was 18.9% (95% CI: 12.9–26.8%), and among males it was 30.5% (95% CI: 23.8–38.1%). STH prevalence did not differ significantly across age groups, whereas a statistically significant difference was observed between sexes (design-adjusted χ^2^-test, *P* = 0.017).

Species distribution varied geographically. *Ascaris* spp. was more prevalent in the East Jaintia Hills (27.1%, 95% CI: 17.3–39.8%), whereas *N. americanus* was predominantly found in Ri Bhoi (18.9%, 95% CI: 8.9–35.8%). *Ancylostoma ceylanicum* was exclusively found in Ri Bhoi (4.2%, 95% CI: 1.1–14.7%).

### STH prevalence in animals.

The prevalence of *Ascaris* spp. in pigs was 64.5% (95% CI: 52.4–74.9%), whereas the prevalence of *N. americanus* and *A. ceylanicum* was 3.2% (95% CI: 0.6–14.8%) and 1.1% (95% CI: 0.1–10.9%), respectively. Among goats, the prevalence of *Ascaris* spp. was 15.2% (95% CI: 7.9–26.9%), whereas *N. americanus* and *A. ceylanicum* were each detected in 3.0% (95% CI: 0.2–28.9%) of samples.

*Ascaris* spp. was isolated from animals across all study villages, similar to its presence in human samples. Co-occurrence of *N. americanus* and *A. ceylanicum* in both humans and animals was observed in three and one village, respectively (Supplemental Figures 1 and 2).

## DISCUSSION

This study provides evidence of continued STH transmission in rural Meghalaya, despite ongoing biannual deworming efforts under the National Deworming Day program, which has targeted children aged 1–19 years since 2016,^13^ The program reported approximately 91% coverage in 2022. Even though the overall STH prevalence observed in this study was relatively low, possibly reflecting the impact of the sustained deworming initiative, as also supported by the high self-reported coverage documented among participants, the continued detection of STH infections in both humans and animals highlights untreated infection reservoirs, which may sustain transmission in endemic communities.

*Ancylostoma ceylanicum* is the second most prevalent hookworm infecting humans in the Asia-Pacific region, after *N. americanus*.^14^ In this study, *A. ceylanicum* was detected in both humans and animals. Dogs are the primary reservoir of *A. ceylanicum*, and its distribution aligns with areas having a high dog population.^14^ In Meghalaya, where stray dog populations are particularly high,^15^ the risk for zoonotic transmission is increased, particularly in areas of poor sanitation and in the absence of routine animal deworming. *Ancylostoma ceylanicum* has been isolated from stray dogs in Tamil Nadu^16^ and Sikkim,^17^ confirming its presence in India, including the NER.

Human STH species, namely *Ascaris* spp. and *N. americanus*, were detected in animals (pigs and goats), highlighting their potential role as zoonotic reservoirs or as mechanical carriers or transport hosts.^18^ Pigs are the most owned livestock animal in Meghalaya. Their coprophagic behavior, coupled with the widespread use of pig feces as manure, may contribute to environmental contamination and sustained STH transmission in areas with high pig density.

This study has several limitations. The small sample size, covering only a few selected districts and villages, may limit the generalizability of the findings. Additionally, some *Ascaris* spp. detected in pigs and humans could potentially represent either *A. suum* (primarily infecting pigs) or *A. lumbricoides* (primarily infecting humans), as the two species form part of a species complex and cannot be distinguished by qPCR.^19^ Moreover, although *A. ceylanicum* was detected in humans, its presence in dogs could not be ascertained because collecting fresh, identifiable samples was difficult.

## CONCLUSION

This study provides insights into the epidemiology of STH in Meghalaya. The detection of *A. ceylanicum* in humans, alongside *Ascaris* spp. and *N. americanus* in animals, across several villages highlights the potential for a complex transmission interface involving humans, animals, and shared environment in settings with high animal density. These findings underscore the need to study STH infections in sympatric populations using a One Health approach to achieve the WHO’s goal for STH control.

## Supplemental Materials

10.4269/ajtmh.25-0401 Supplemental Materials
