# Supplementary material for: Zoonotic Transmission of Soil-Transmitted Helminths in a Setting with Close Human–Animal Interaction: A Cross-Sectional Pilot Study from Meghalaya, India
Source: Am J Trop Med Hyg. 2025 Nov 11;114(1):138–41. doi: 10.4269/ajtmh.25-0401 (PMC12781455; doi:10.4269/ajtmh.25-0401)
Supplement: Supplemental Materials [file tpmd250401.SD1.pdf]

Supplementary Materials

Text S1: Details of the species specific-primer sequences and qPCR procedure:

"The qPCR assay details and species- specific primer sequences used for the detection of *N. americanus*, and *T. trichiura* are published in Pilotte N, 2016[1], *A. ceylanicum* in Papaiakovou M, 2017[2], *A. lumbricoides/A. suum* in Pilotte N, 2019[3], and *A. caninum* is yet to be published (developed by Dr. Steven William's lab, Smith College, Northampton, MA). All these assays used a small-volume reaction setup (7uL total reaction volume) that targets the non-coding repetitive sequences of the species genome using double-quenched probes."

References:

1. Pilotte N, Papaiakovou M, Grant JR, Bierwert LA, Llewellyn S, McCarthy JS, *et al*. 2016. Improved PCR-Based Detection of Soil Transmitted Helminth Infections Using a Next-Generation Sequencing Approach to Assay Design. *PLoS Negl Trop Dis*. 10: e0004578.

2. Papaiakovou M, Pilotte N, Grant JR, Traub RJ, Llewellyn S, McCarthy JS, *et al.* 2017. A novel, species-specific, real-time PCR assay for the detection of the emerging zoonotic parasite Ancylostoma ceylanicum in human stool. *PLoS Negl Trop Dis*. 11: e0005734.

3. Pilotte N, Maasch JRMA, Easton AV, Dahlstrom E, Nutman TB, Williams SA. 2019. Targeting a highly repeated germline DNA sequence for improved real-time PCR-based detection of Ascaris infection in human stool. *PLoS Negl Trop Dis*. 13: e0007593.

Fig S1: Survey-weighted estimates of STH prevalence in humans by study villages

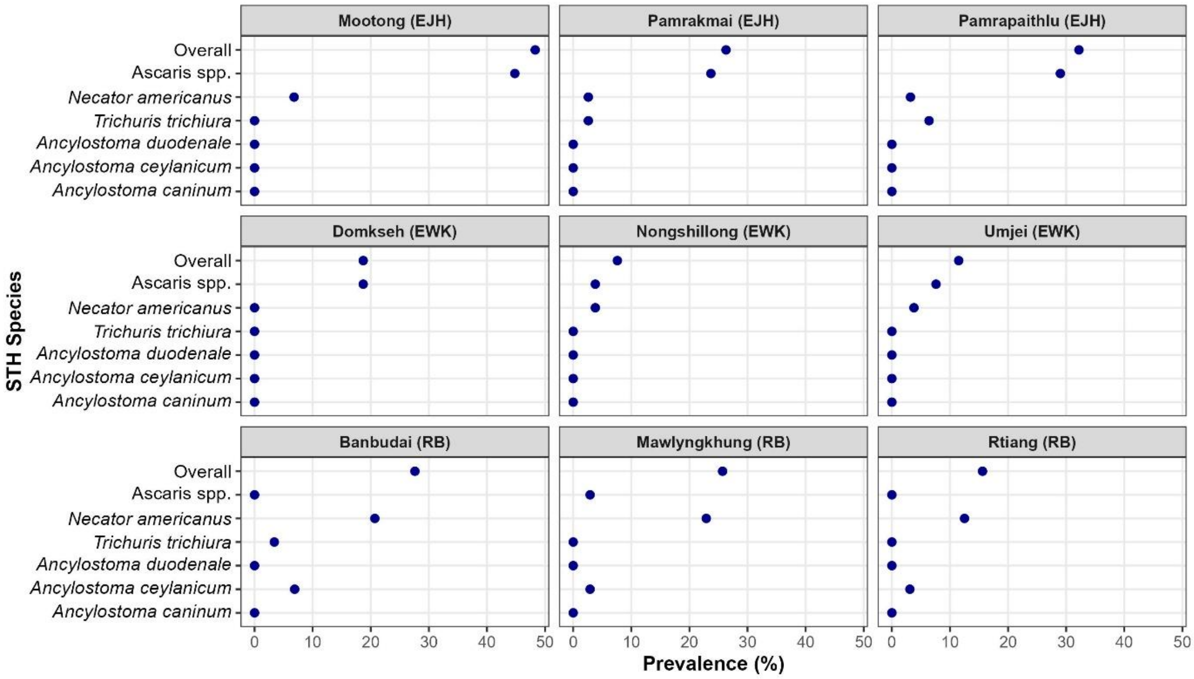

EJH: East Jaintia Hills District; EWK: Eastern West Khasi Hills District; RB: Ri Bhoi District

Fig S2: Unweighted estimates of STH prevalence in animals by study villages

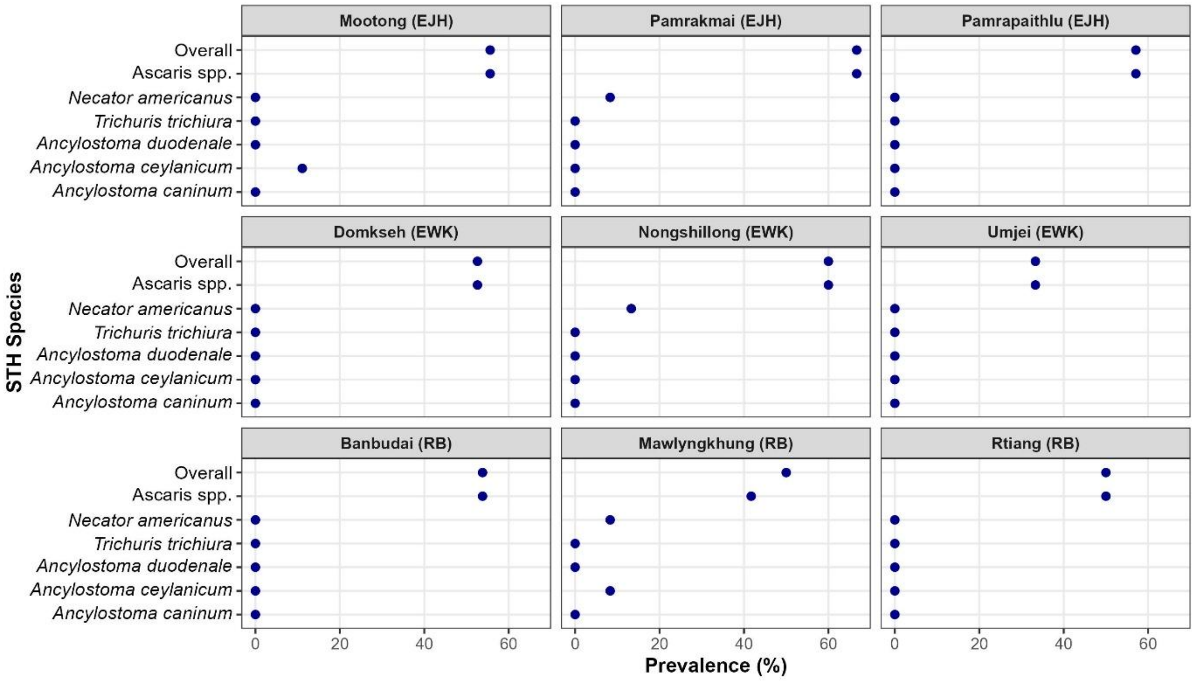

EJH: East Jaintia Hills District; EWK: Eastern West Khasi Hills District; RB: Ri Bhoi District
